# Knowledge, attitudes, and practices of seasonal influenza vaccination among older adults in nursing homes and daycare centers, Honduras

**DOI:** 10.1371/journal.pone.0246382

**Published:** 2021-02-11

**Authors:** Zachary J. Madewell, Rafael Chacón-Fuentes, Jorge Jara, Homer Mejía-Santos, Ida-Berenice Molina, Juan Pablo Alvis-Estrada, Raul Espinal

**Affiliations:** 1 Centro de Estudios en Salud, Universidad del Valle de Guatemala, Guatemala City, Guatemala; 2 Unidad de Vigilancia de la Salud, Secretaría de Salud de Honduras, Tegucigalpa, Honduras; 3 Programa Ampliado de Inmunizaciones, Secretaría de Salud de Honduras, Tegucigalpa, Honduras; 4 Dirección General del Adulto Mayor, Secretaria de Estado en los Despachos del Interior y Población de Honduras, Tegucigalpa, Honduras; Waikato Institute of Technology, NEW ZEALAND

## Abstract

**Background:**

Older adults represent 70–90% of seasonal influenza-related deaths and 50–70% of influenza-related hospitalizations. Vaccination is the most efficient means of preventing influenza and reducing influenza-related illnesses. We aimed to describe knowledge, attitudes, and practices (KAP) of seasonal influenza vaccination among older adults in Honduras.

**Methods:**

From August 29–October 26, 2018, we conducted a cross-sectional KAP survey regarding seasonal influenza vaccinations to samples of older adults 1) admitted to nursing homes and 2) attending daycare centers. We used the Minimental State Examination (MMSE) psychometric tool to assess the cognitive status of older adults and included participants with scores of ≥23 points in the survey. We reported frequency distributions for demographics, KAP of influenza virus and vaccination, and vaccination coverage. We used logistic regression to analyze associations between demographics and verified influenza vaccination.

**Results:**

Of 511 MMSE participants, 341 completed the survey (95 adults in 12 nursing homes and 246 older adults in ten daycare centers). Almost all participants knew that influenza causes severe illness and may be transmitted from person to person, vaccination is safe and protects against disease, and older adults have greater risk of complications. Of 284 participants with verified vaccinations, 81.3% were vaccinated for influenza: 87.9% attending daycare centers and 61.4% in nursing homes. Among all participants, verified current influenza vaccination was associated with self-reported influenza vaccination in previous year (aOR: 14.05; 95% CI: 5.36–36.81); no formal education (aOR: 4.83; 95% CI: 1.63–14.37) or primary school education (aOR: 4.51; 95% CI: 1.79–11.37) having ≥secondary as reference; and indigenous (aOR: 4.55; 95% CI: 1.18–17.49) having Mestizo as reference. Reasons for vaccination were perceived self-benefits, protection against influenza complications, favorable vaccination hours, and healthcare provider recommendations.

**Conclusion:**

Four-fifths of older adults were vaccinated for seasonal influenza. Educational efforts provided in conjunction with vaccination campaigns resulted in high knowledge of influenza virus, transmission, and vaccination. Further outreach regarding disease risks and vaccine safety needs to be directed towards older adults in nursing homes who had lower knowledge and coverage than older adults in daycare centers.

## Introduction

Influenza is an acute infectious disease that affects 5–10% of the world’s adult population and is responsible for 3–5 million cases of severe illness and 290,000–650,000 deaths worldwide annually [1, 2]. Approximately half of influenza infections are asymptomatic or mild, but influenza may cause severe complications including acute upper respiratory tract infections, pneumonia, and cerebrovascular events, particularly among adults ≥65 years of age [3]. Older adults (≥65 years) are at increased risk of influenza-related complications, hospitalization, and mortality, particularly those with underlying chronic diseases [4]. They represent 70–90% of seasonal influenza-related deaths and 50–70% of influenza-related hospitalizations from complications including pneumonia, myocarditis and encephalitis [2]. This study was conducted in Honduras, where diseases of the respiratory system account for 10% of all deaths [5]. The most common causes of hospitalization for respiratory infections in Honduras are pneumonia, bronchiolitis, and asthma, which together account for 8% of healthcare expenditures [6]. From 2011–2015, the influenza-related hospitalization rate for adults ≥60 years in Honduras was 16.1 per 100,000 population (95% CI: 15.0–17.2) and influenza-related mortality was 1 per 100,000 population (95% CI: 0.7–1.3) [7].

Seasonal influenza vaccination is the most efficient means of preventing influenza and reducing influenza-related illnesses [8]. In addition to preventing influenza, vaccination decreases morbidity from influenza and pneumonia, risk of hospitalization, and severe respiratory infections or cardiopulmonary complications [9]. World Health Organization (WHO) Strategic Advisory Group of Experts on Immunization considers adults ages ≥65 years to be among the target groups for influenza vaccines and urges vaccination each year [2]. Despite these recommendations, coverage is low among older adults worldwide [10]. Reasons cited in other studies for non-vaccination for seasonal influenza among older adults include safety concerns, lack of knowledge, poor understanding of adverse health outcomes, and belief that vaccination causes influenza [10–12]. Conversely, factors associated with vaccination include pressure from family and friends, previous positive experiences, and frequent interactions with healthcare systems [11, 13, 14].

In Central American countries, influenza epidemics typically start in May or June and last an average of four to fifth months [15, 16]. Individuals of high risk groups (e.g., children <5 years, older adults, pregnant women, people with underlying high-risk conditions, healthcare workers) are vaccinated for seasonal influenza during annual vaccination campaigns, which are conducted from April through June [17, 18]. With the exception of Belize and Guatemala, the composition vaccine for the Southern Hemisphere is used during these campaigns when the most updated formulation is available [19]. However, influenza vaccination is not used consistently across Central American countries. In 2018, seasonal influenza vaccination coverage among older adults ranged from 41% in Belize to 100% in Panama [20].

The Ministry of Health of Honduras (SESAL) launched the Expanded Program of Immunization (EPI) in 1979 with the aim of achieving universal vaccination coverage for a number of vaccine-preventable diseases, including seasonal and pandemic influenza, through mass vaccination, epidemiological surveillance, educational activities, and social participation [21]. EPI began vaccination campaigns for seasonal influenza in 2003 with the goal of achieving ≥95% coverage for prioritized risk groups (pregnant women, children 6 months to five years of age, adults >60 years of age, people with chronic diseases, healthcare workers, poultry farm workers) [22]. The Vaccine Law of the Republic of Honduras mandates that all residents, including older adults, be vaccinated for all vaccine-preventable diseases determined by SESAL, which includes influenza [23]. Influenza vaccines are available free-of-charge in public health centers and other healthcare facilities of the Honduran Social Security Institute (IHSS) nationwide [24].

Better understanding of factors influencing seasonal influenza vaccination among older adults can be used to guide targeted interventions to improve coverage for this risk group. We therefore aim to describe KAP for vaccination of seasonal influenza among older adults in Honduras.

## Materials and methods

### Study design

We conducted a cross-sectional KAP survey regarding seasonal influenza vaccinations to a sample of older adults 1) admitted to nursing homes and 2) attending daycare centers and supervised by the General Directorate for the Elderly (DGAM) of the Secretary of State in the Government, Justice and Decentralization Offices.

### Study setting

Honduras has an area of 112,492 km^2^ and is divided administratively into 18 departments (political subdivisions similar to U.S. states with governors appointed by the President of Honduras) and 298 municipalities [25]. The total population is 9,746,000 of which 7.4% are ≥60 years or retired (female: 287,000; male: 256,000) [26, 27]. The older adults population is anticipated to grow at a rate of 3.9% from 2020–2025, three times faster than the total population [28]. The average life expectancy in Honduras is 71.3 years (female: 73.0 years; male: 69.6 years) and death rate is 5.3 deaths per 1,000 population [25]. Of older adults with some education, 85% have completed primary school, 12% secondary school, and 3.4% higher education [29]. Of all older adults in Honduras, 7.5% have a pension or retirement benefits [30].

The health sector in Honduras consists of public and private sectors. The public sector includes SESAL and IHSS [5]. SESAL serves the entire Honduran population, but only 50–60% of Hondurans use these services [5]. IHSS covers 40% of employed individuals and their dependents [5]. Approximately 17% of the population does not have regular access to healthcare services [5]. Per capita healthcare expenditure was $212 in 2014 or 7.6% of the total gross domestic product [25]. DGAM, supported by Pan American Health Organization (PAHO), was established in 2008 to ensure compliance with the Comprehensive Law on the Protection of the Elderly and respect for the rights of 742,500 older and retired adults nationwide [30].

### Questionnaire

We adapted a questionnaire from the Centers for Disease Control and Prevention (CDC) influenza survey [31] and previous experience from another KAP study of older adults in Costa Rica [32]. The questionnaire was modified following an evaluation of technical detail and cultural appropriateness by personnel from the Section of Psychological Attention of DGAM and by the Institutional Review Boards of Universidad del Valle de Guatemala (UVG) and Universidad Nacional Autónoma de Honduras (UNAH). We pilot-tested the questionnaire and informed consent with the patient care staff of one of the elderly care centers and with 34 older adults from one of the outpatient care centers four weeks before study implementation. Modifications were made to the language and order of questions included in the questionnaire based on responses by the participants. The finalized questionnaire included demographics (sex, age, department of residence, education, race, marital status, concurrent chronic disease), knowledge of benefits and risks of influenza vaccination, influenza vaccination status, reasons for and for not receiving influenza vaccination, perceived risk of influenza, and clinical manifestations following vaccination (S1 Questionnaire).

We conducted close-ended surveys from August 29 to October 26, 2018, three months after the launch of the influenza vaccination campaign of Honduras on May 14, 2018. We administered surveys in nursing homes and daycare centers supervised by the DGAM of the Secretary of State in the Government, Justice and Decentralization Offices. Surveys were done by interviews in Spanish and data collected with tablets, using the Research Data Management Center application (Open Data Kit ODK JAVA). Interviewers were healthcare professionals trained in relevant aspects of influenza vaccination. We reviewed vaccination cards and medical records to confirm vaccination status and determine whether there were other risk factors for complications related to influenza virus [33].

### Study population

To calculate the sample size of adults admitted to nursing homes or attending daycare centers, we used the lowest administrative vaccination coverage for influenza among older adults in Central American countries reported by PAHO in 2015 as a key indicator: 83% [20]. The number of older adults in nursing homes and supervised by the DGAM of Honduras (869 people) and in daycare centers (1,107 people) served as the reference population [30]. We used a design effect of 1.5, because the sites were selected in two stages: first the random selection of the establishments in each of the regions, and then the random selection of participants except in small sites where we attempted to include all possible participants. We also used a replacement rate of 10%, which was based on previous experience from another KAP study of older adults in Costa Rica [32]. Applying 5% accuracy and 95% confidence interval, we calculated sample sizes of 245 older adults admitted to nursing homes and 312 attending daycare centers (S1 File).

**Fig 1:**
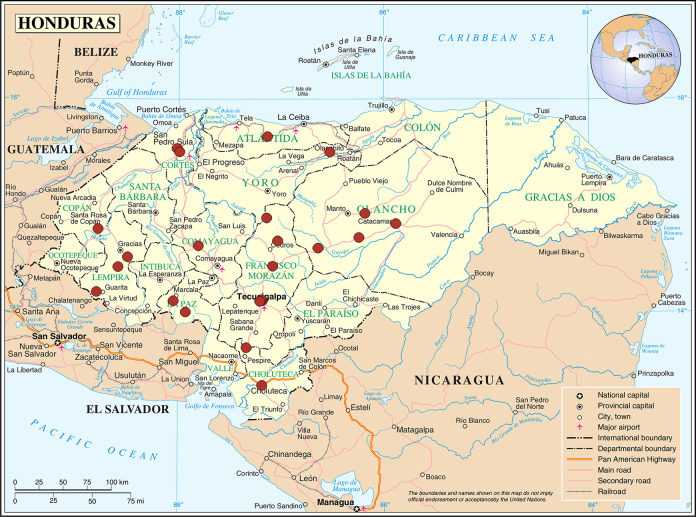
Location of the 22 daycare centers and nursing homes, study of knowledge, attitudes and practices of seasonal influenza vaccination, older adults, Honduras, 2018. Source: This map is a public domain file provided by the Central Intelligence Agency’s World Factbook, https://www.cia.gov/library/publications/the-world-factbook/.

We used separate probabilistic, two-stage, stratified and conglomerate sampling to select samples of persons: (1) admitted to nursing homes and supervised by the DGAM; and (2) attending daycare centers and supervised by the DGAM. Stratification was based on nursing home or daycare center locations (West, Northeast, and Central). In stage one, we identified conglomerates (nursing homes or daycare centers) in each stratum by probability proportional to the number of admitted adults. In stage two, we invited all adults ≥60 years in each selected conglomerate who were available between August 29 and October 26, 2018. Participants admitted to nursing homes were located in seven of the 18 departments of Honduras and participants attending daycare centers were located in six departments (Fig 1).

We used the Minimental State Examination (MMSE) psychometric tool MEC-35 [34] to assess the cognitive status of older adults. We invited all adults ages ≥60 years admitted to nursing homes or attending daycare centers supervised by the DGAM from August 1 to October 8, 2018 with a MMSE test score of ≥23 points to participate. Excluded participants were those who could not respond to interview questions because of their clinical condition, nonresidents of Honduras, and those with cognitive impairment evidenced by a MMSE score of <23 points. These latter participants were not considered appropriate to include based on a recommendation from DGAM.

### Ethics statement

This study was approved by the Research Ethics Committee of UVG (Protocol number 173-10-2017), Bioethics Committee of UNAH (study code 2018011), and Teaching and Research Department of IHSS. We obtained written informed consent for all participants.

### Statistical analysis

We reported frequency distributions for sex, age, and MMSE results for older adults who participated in the MMSE. We used Pearson Chi-square tests to evaluate associations between sex, age, and MMSE scores, and recruitment location (nursing home, daycare center).

We reported frequency distributions of demographic variables (sex, age group, department of residence, education, race, marital status, concurrent chronic disease, self-reported influenza vaccination status in previous year [2017], self-reported and verified current [2018] influenza vaccination) for participants included in KAP surveys. We reported frequencies, proportions, and 95% confidence intervals (CI) of knowledge of influenza vaccination, reasons for and for not receiving the vaccination, and clinical manifestations within seven days of vaccination. We reported results for all participants and results stratified by recruitment location (nursing home, daycare center) to evaluate differences in KAP determinants between these settings. We used Pearson Chi-square tests to evaluate associations between demographics and KAP of influenza vaccination, and recruitment location (nursing homes, daycare centers).

We used logistic regression to analyze associations between demographics and verified current influenza vaccination for 1) all participants, 2) participants in nursing homes, and 3) participants in daycare centers. These analyses excluded those with unverified influenza vaccinations and those who did not provide complete demographics. Statistical significance was evaluated through the Wald Chi-square test. Variables found to be significant at *P*<0.20 from bivariate analyses were included in manual forward step-wise multivariable logistic regression models to evaluate associations with influenza vaccination. Variables with the smallest *P-*value from bivariate analyses were added one at a time to the forward step-wise regression models and removed at a *P<*0.20 significance level. Values of *P*<0.05 were considered statistically significant. We used tolerance values to assess collinearity among all independent variables and Hosmer-Lemeshow to assess goodness-of-fit of the final adjusted model. We report unadjusted and adjusted odds ratios, and 95% CIs. We used SAS V.9.4 (SAS Institute, Inc., Cary, North Carolina) for all analyses.

## Results

### Sample characteristics

Of 511 older adults who completed the MMSE, 348 had scores within normal ranges (≥23 points). Five older adults were unable to interview due to logistical problems and two refused to participate, therefore 341 completed surveys (S1 Table). MMSE scores for adults attending daycare centers were significantly higher than those admitted to nursing homes (p<0.001).

We surveyed 95 older adults in 12 nursing homes and 246 in ten daycare centers (Table 1). The most prevalent departments of residence were Francisco Morazán (31.1%), Olancho (17.6%), and Lempira (17.3%). The median age of adults in nursing homes and daycare centers was 76 years (interquartile range: 68–84 years) and 70 years (interquartile range: 65–77 years), respectively. Of all participants, 54.3% were female, 60.3% had primary school education, 72.7% were Mestizo (mixed Amerindian and European ancestry), 30.5% were married, and 59.5% had a concurrent chronic disease. A greater proportion of participants in nursing homes were older and had a concurrent chronic disease compared than those in daycare centers (*P*-values<0.001) (S2 Table).

**Table 1:** Demographics of 341 older adults in nursing homes and daycare centers^a^, Honduras, August 29 to October 26, 2018.

Characteristic	n (%)
Female sex	185 (54.3)
Age (in years)	
65–70	156 (45.8)
71–80	115 (33.7)
≥81	70 (20.5)
Department of Residence	
Francisco Morazán	106 (31.1)
Olancho	60 (17.6)
Lempira	59 (17.3)
La Paz	52 (15.2)
Choluteca	29 (8.5)
Other	35 (10.3)
Education (n = 330)	
No formal education	86 (26.1)
Primary	199 (60.3)
≥Secondary	45 (13.6)
Race	
Mestizo	248 (72.7)
Indigenous	69 (20.2)
Other	24 (7.1)
Marital status	
Married	104 (30.5)
Single	88 (25.8)
Accompanied	34 (10.0)
Separated/divorced	33 (9.7)
Widowed	82 (24.0)
Concurrent chronic disease^b^	203 (59.5)
Self-reported influenza vaccination in previous year (n = 331)^c^	289 (87.3)
Self-reported current influenza vaccination (n = 329)^c^	276 (86.0)
Verified^d^ current influenza vaccination (n = 284)^e^	231 (81.3)

^a^ See S2 Table for results stratified by recruitment location.

^b^ 57 chronic heart disease, 42 diabetes mellitus, 14 asthma, 9 bronchitis, 8 chronic obstructive pulmonary disorder, 8 cerebrovascular disease, 3 cancer, 2 chronic kidney disease, 136 other disease.

^c^ Excluded participants who did not know if they were vaccinated.

^d^ Verified with vaccination cards and medical records.

^e^ Excluded 12 participants who did not know if they were vaccinated and 45 who professed vaccinations without verification.

### Knowledge of influenza vaccination

Almost all participants knew the influenza vaccine is safe (95.5%; 95% CI: 93.2–97.7%) and protects against disease (96.6%; 95% CI: 94.6–98.6%); influenza virus may be transmitted from person to person (95.4%; 95% CI: 93.2–97.7%) or via contaminated hands (94.9%; 95% CI: 92.6–97.3%); influenza may cause serious illness (96.7%; 95% CI: 94.7–98.6%); and older adults have greater risk of complications resulting from infection (97.9%; 95% CI: 96.3–99.4%) (Table 2). A greater proportion of participants in daycare centers knew that influenza virus causes serious illness and may be transmitted from person to person or via contaminated hands, and the influenza vaccine is safe and protects against disease than those in nursing homes (*P*-values≤0.024) (S3 Table).

**Table 2:** Knowledge of influenza vaccination, older adults in nursing homes and daycare centers^a^, Honduras, August 29 to October 26, 2018.

Knowledge	Total^b^ n	Agreedn	Agreed % (95% CI)
Influenza causes severe illness	329	318	96.7 (94.7–98.6)
Older adults have a higher risk of complications from influenza	332	325	97.9 (96.3–99.4)
Influenza may be transmitted from person to person	328	313	95.4 (93.2–97.7)
Influenza may be transmitted if people touch their mouths or noses with contaminated hands	335	318	94.9 (92.6–97.3)
Aware of an influenza vaccine	332	329	99.1 (98.1–100)
The vaccine protects against influenza complications	324	313	96.6 (94.6–98.6)
Perceived vaccine as safe	331	316	95.5 (93.2–97.7)

CI: confidence interval.

^a^ See S3 Table for results stratified by recruitment location.

^b^ Excluded participants who did not respond.

### Influenza vaccination

Of 284 older adults whose vaccination records were available and verified, 231 (81.3%; 95% CI: 76.8–85.9%) were vaccinated for influenza, including 43 (61.4%; 95% CI: 49.7–73.1%) in nursing homes and 188 (87.9%; 95% CI: 83.4–92.3%) in daycare centers (Table 2).

The final model for verified current influenza vaccination included education, race, and self-reported influenza vaccination in previous year (Table 3). Adjusting for the other variables in the model, the odds of verified current influenza vaccination were 14.05 times higher for older adults who self-reported influenza vaccination in the previous year (95% CI: 5.36–36.81); 4.83 and 4.51 times higher for those with no formal education (95% CI: 1.63–14.37) and primary school education (95% CI: 1.79–11.37), respectively, having ≥secondary as reference; and 4.55 times higher for indigenous participants (95% CI: 1.18–17.49) having Mestizo as reference. The Hosmer-Lemeshow goodness-of-fit test demonstrated the model fit was adequate (p = 0.93). Tolerance values for all independent variables were >0.99, so there was no evidence of collinearity. Results restricted to participants in nursing homes were similar, except that race was not associated with current influenza vaccination (S4 Table). For participants attending daycare centers, only self-reported influenza vaccination in previous year was associated with current influenza vaccination (S5 Table).

**Table 3:** Associations between demographics and influenza vaccination (verified^a^), older adults in nursing homes and daycare centers^b^ (n = 267)^c^, Honduras, August 29 to October 26, 2018.

Variable	OR (95% CI)	p-value	aOR^c^ (95% CI)	p-value
Female sex (Ref: male)	1.09 (0.58–2.06)	0.793	–	–
Age (Ref: ≥81 years)		0.390		–
≤70 years	1.74 (0.78–3.90)		–	
71–80 years	1.51 (0.67–3.43)		–	
Education (Ref: ≥secondary)		<0.001		0.003
No formal education	5.83 (2.19–15.52)		4.83 (1.63–14.37)	
Primary incomplete or complete	5.30 (2.34–12.04)		4.51 (1.79–11.37)	
Race (Ref: Mestizo)		0.030		0.035
Indigenous	4.55 (1.35–15.35)		4.55 (1.18–17.49)	
Other	0.65 (0.22–1.92)		0.50 (0.15–1.64)	
Marital status (Ref: Single)		0.087		–
Married	3.41 (1.39–8.36)		–	
Accompanied	2.15 (0.66–7.01)		–	
Separated / divorced	1.15 (0.37–3.56)		–	
Widowed	1.86 (0.79–4.39)		–	
Concurrent chronic disease (Ref: no)	1.14 (0.60–2.15)	0.700	–	–
Self-reported influenza vaccination in previous year	11.19 (4.77–26.24)	<0.001	14.05 (5.36–36.81)	<0.001

Ref: reference; OR: odds ratio; aOR: adjusted odds ratio; CI: confidence interval.

^a^ Verified with vaccination cards and medical records.

^b^ See S4 Table for results restricted to participants in nursing homes and S5 Table for participants in daycare centers.

^c^ Analyses excluded participants with unverified influenza vaccinations in 2018, those who did not respond to educational attainment, and those who did not know their vaccination status in 2017.

^d^ Adjusted for the other variables listed in the model.

### Reasons for and for not receiving influenza vaccination

Of 231 participants who were vaccinated for seasonal influenza, 205 cited perceived self-benefits (88.7%; 95% CI: 84.6–92.9%), 150 cited favorable vaccination hours (64.9%; 95% CI: 58.7–71.1%), 144 cited protection from influenza complications (62.3%; 95% CI: 56.0–68.6%), and 143 cited healthcare provider recommendations (61.9%; 95% CI: 55.6–68.2%) as reasons for vaccination (Table 4). A greater proportion of participants from daycare centers cited favorable vaccination hours, perceived personal risk, protection from influenza complications, costs of treatment for illness, doctor and family recommendations, mandatory vaccination policy, and mass media as reasons for vaccination than participants in nursing homes, whereas fewer professed being offered the vaccine (p-values≤0.043) (S6 Table).

**Table 4:** Reasons for receiving influenza vaccination, older adults in nursing homes and daycare centers^a^, Honduras, August 29 to October 26, 2018.

	All vaccinated participants (n = 231)	
Reason	Agreed n	% (95% CI)
*Easy access*		
Offered the vaccine at nursing home or daycare center	122	52.8 (46.3–59.3)
Favorable vaccination hours	150	64.9 (58.7–71.1)
*Perceived benefits*		
Perceived self-benefits of vaccination	205	88.7 (84.6–92.9)
Vaccine protects from complications	144	62.3 (56.0–68.6)
Perceived personal risk for influenza	105	45.5 (39.0–51.9)
Vaccination protects peers	37	16.0 (11.3–20.7)
Mild side effects perceived better than contracting influenza	101	43.7 (37.3–50.2)
To negate costs of treatment for influenza	135	58.4 (52.0–64.8)
*Previous experiences*		
No problems with previous vaccination	127	55.0 (48.5–61.4)
Have not observed negative effects of vaccination	54	23.4 (17.9–28.9)
*Peer influence*		
Peers recommended vaccination	53	22.9 (17.5–28.4)
Knowledge that the majority of peers get vaccinated	44	19.0 (13.9–24.1)
Peers expected vaccination	26	11.3 (7.1–15.4)
Urged to get vaccinated by family members	66	28.6 (22.7–34.4)
*Health establishment counseling*		
Was informed vaccination is mandatory	79	34.2 (28.0–40.4)
Urged to get vaccinated by a doctor or nurse	143	61.9 (55.6–68.2)
Listened to promotional outreach on vaccinations at nursing home or daycare center	46	19.9 (14.7–25.1)
*Aware of vaccine benefits from mass media*	68	29.4 (23.5–35.4)

CI: confidence interval.

^a^ See S6 Table for results stratified by recruitment location.

Among 53 unvaccinated participants, reasons provided for non-vaccination were heterogeneous and included dissuasion by family members, fear of contracting influenza, and not being offered the vaccine (Table 5).

**Table 5:** Reasons for not receiving influenza vaccination, older adults in nursing homes and daycare centers (n = 53), Honduras, August 29 to October 26, 2018.

Reasons	n	% (95% CI)
*Fear of adverse effects*		
Fear of contracting influenza	4	7.5 (0.2–14.9)
Fear of side effects	1	1.9 (0–5.7)
Fear of pain caused by the needle	3	5.7 (0–12.1)
*Confidence in the vaccine*		
Belief vaccine does not prevent disease	1	1.9 (0–5.7)
Belief vaccine does not protect peers	3	5.7 (0–12.1)
*Limited access*		
Vaccine not available	3	5.7 (0–12.1)
Was not instructed to be vaccinated	3	5.7 (0–12.1)
Was not offered the vaccine	4	7.5 (0.2–14.9)
Did not know where to go for vaccine	1	1.9 (0–5.7)
*Influence of family and friends*		
Vaccine not accepted by nursing home or daycare center peers	1	1.9 (0–5.7)
Family members said not to get vaccinated	5	9.4 (1.3–17.6)

CI: confidence interval.

### Clinical manifestations of vaccination

Of 231 participants who were vaccinated for influenza in 2018, 55 (23.8%; 95% CI: 18.3–29.3%) reported mild or moderate untoward reactions after vaccination, including vaccination site pain, flu-like symptoms, and fever (Table 6).

**Table 6:** Clinical manifestations seven days after vaccination, older adults in nursing homes and daycare centers (n = 231), Honduras, August 29 to October 26, 2018.

Clinical manifestation	n	% (95% CI)
Pain at the vaccination site	31	13.4 (9.0–17.8)
Flu-like symptoms	15	6.5 (3.3–9.7)
Fever	14	6.1 (3.0–9.2)
General discomfort	11	4.8 (2.0–7.5)
Inflammation at the vaccination site	3	1.3 (0–2.8)
Dizziness	1	0.4 (0–1.3)

CI: confidence interval.

## Discussion

Seasonal influenza vaccination coverage in older adults was 81.3% with higher coverage among those treated in daycare centers (87.9%) than residents in nursing homes (61.4%). Coverage in this study was slightly lower than that reported by PAHO for older adults in Honduras (88%), Panama (100%), and Costa Rica (92%) in 2017 [20]. Seasonal influenza vaccination coverage rates for older adults in Central America are among the highest worldwide [35, 36]. Coverage in our study falls short of the herd immunity threshold for older adults in the United States (90%), but exceeds the European threshold of 75% [37].

Knowledge of influenza transmission and vaccination was high, which is consistent with other studies of older adults [13, 38]. High knowledge and coverage of influenza among older adults in Honduras may be attributed to SESAL’s robust EPI, which is supported by WHO, PAHO, and United Nations Children’s Fund [39], and aims to vaccinate >500,000 older adults for seasonal influenza during vaccination campaigns [40]. Our study was conducted three months after the launch of vaccination campaigns, which include educational activities for patients and healthcare providers that may have improved the participants’ knowledge.

Adults in daycare centers had higher knowledge of influenza virus, transmission, and vaccination; and higher seasonal influenza vaccination coverage than adults in nursing homes. A greater proportion of vaccinated adults in daycare centers cited favorable vaccination hours, family member and healthcare provider recommendations, Honduras’s mandatory vaccination policy, costs of treatment for illness, and mass media as reasons for vaccination than adults in nursing homes. Higher coverage among older adults in daycare centers than nursing homes may be due to the ease of assembling adults into groups for vaccination campaigns in daycare centers. Greater vigilance, recordkeeping, and outreach might be required in nursing homes in light of the apparent cognitive decline of this largely non-ambulatory population.

Influenza vaccination in the previous year had the strongest association with current seasonal influenza vaccination for participants in nursing homes and daycare centers, which is in accord with another study of older adults in the United States [41]. Vaccinated older adults profess greater intention to get vaccinated the following year [41, 42].

Although higher educational attainment is often associated with greater seasonal influenza vaccination uptake among community-dwelling older adults [14, 38, 43], our study demonstrated that participants with higher education were less likely to be vaccinated for influenza than adults with primary or no formal education. Among unvaccinated participants with ≥secondary education, reasons for non-vaccination included family dissuasion, fear of contracting influenza, and belief that vaccination is ineffective. Perhaps the higher educated group is more influenced by anti-vaccination campaigns on social media networks to which the lower educated groups are less likely to be exposed [11, 44]. Adverse side effects of seasonal influenza vaccination are rare, but tend to be sensationalized by the media [45]. Our findings are consistent with a study in Beijing, which showed that older adults with lower education were more likely to recognize influenza as a serious illness and follow instructions from healthcare workers, and less likely to report fear of side effects than those with higher educational attainment [11]. In addition to promoting information regarding the safety and efficacy of influenza vaccinations, education is needed to dispel misconceptions concerning vaccination side effects. Future vaccination campaigns should tailor communication strategies to older adults.

The finding that indigenous participants were more likely to be vaccinated than Mestizos may be attributed in part to the National Health Model in Honduras, which has attempted to increase healthcare access for 1.4 million people living in remote and impoverished areas via the deployment of 367 primary healthcare teams that work to promote qualitative improvements in health and behavior, and provide preventive treatment [5, 46]. We did not find associations between other demographics (sex, age, marital status, concurrent chronic disease) and seasonal influenza vaccination. These findings are in contrast to other studies that identified advanced age [13], female sex [47], married [48, 49], and concurrent chronic diseases [11, 47] as important predictors of vaccination among older adults.

The most cited reasons for vaccination were perceived benefits, favorable vaccination hours, healthcare worker and peer recommendations, and previous positive experiences, which are consistent with the literature [11–14]. Older adults who receive direct recommendations from healthcare personnel are less likely to be concerned of side effects, which may increase vaccination uptake [10, 11]. These findings underscore the value of healthcare worker recommendations to seniors during their frequent interactions. This tutelage may highlight vaccine efficacy and safety, older adult susceptibility to influenza, and complications of influenza.

Reasons cited for non-vaccination including not being offered the vaccine, not knowing it was necessary to be vaccinated, and concerns of side effects are consistent with other studies [10, 12, 50]. Reasons cited for non-vaccination by older adults in other studies such as infrequent contact with the healthcare system, transportation inconvenience, and high costs [10–12, 51] were not barriers for vaccination in this study. Methods to improve vaccination among older adults may include express vaccination clinics [52] and nursing home visits [53], which could reduce access barriers. Postcard reminders and information pamphlets may also increase influenza vaccination among older adults [54].

This study had several limitations. First, this study included older adults admitted to nursing homes or attending daycare centers as well as those with adequate cognitive levels as evidenced by the MMSE, and may therefore not be representative of all older adults in Honduras. Second, this was a cross-sectional study, which precludes establishing causal and temporal relationships between KAP regarding influenza and vaccination behavior. Third, there may have been social desirability bias in responses regarding KAP of influenza virus and vaccinations. Fourth, there may have been differential recall bias if there were differences in subject recall or reporting between vaccinated and unvaccinated participants. Fifth, there may have been response bias if respondents differed from those who declined to participate. Sixth, results from logistic regression analyses should be interpreted with caution due to the wide confidence intervals.

Notwithstanding these limitations, our study included samples of older adults in both nursing homes and daycare centers. Second, MMSE results demonstrated that most older adults had adequate cognitive ability. Third, we were able to verify most of the influenza vaccinations with vaccination cards and medical records. Fourth, to our knowledge this is the first KAP study regarding seasonal influenza vaccination among older adults in Central America.

Four-fifths of older adults were vaccinated for seasonal influenza. Educational efforts provided in conjunction with vaccination campaigns resulted in high knowledge of influenza virus, transmission, and vaccination. Perceived benefits of vaccination and easy access were the most cited reasons for vaccination. Vaccination in the previous year had the strongest association with current vaccination, but we found an inverse association between educational attainment and vaccination. Further outreach regarding disease risks and vaccine safety needs to be directed towards older adults in nursing homes who had lower knowledge and coverage than older adults in daycare centers. Educational activities should be culturally sensitive and appropriate for age and literacy levels. Healthcare workers play a vital role in providing succinct and comprehensive information about vaccines and dispelling misconceptions to older adults.

## Supporting information

S1 Table Demographics of older adults who participated in the Minimental State Examination (n = 511), Honduras, August 29 to October 26, 2018. (DOCX)

S2 Table Demographics of 341 older adults stratified by recruitment location, Honduras, August 29 to October 26, 2018. (DOCX)

S3 Table Knowledge of influenza vaccination stratified by recruitment location, older adults, Honduras, August 29 to October 26, 2018. (DOCX)

S4 Table Associations between demographics and influenza vaccination (verified), older adults in nursing homes (n = 63), Honduras, August 29 to October 26, 2018. (DOCX)

S5 Table Associations between demographics and influenza vaccination (verified), older adults in daycare centers (n = 204), Honduras, August 29 to October 26, 2018. (DOCX)

S6 Table Reasons for receiving influenza vaccination stratified by recruitment location, older adults, Honduras, August 29 to October 26, 2018. (DOCX)

S1 Questionnaire (DOCX)

S1 File Equation used to obtain sample sizes for surveys of older adults. (DOCX)
