# Supplementary material for: Knowledge, attitudes, and practices of seasonal influenza vaccination among older adults in nursing homes and daycare centers, Honduras
Source: PLoS One. 2021 Feb 11;16(2):e0246382. doi: 10.1371/journal.pone.0246382 (PMC7877760; doi:10.1371/journal.pone.0246382)
Supplement: S1 Table — (DOCX) [file pone.0246382.s001.docx]

| **S1 Table. Demographics of older adults who participated in the Minimental State Examination^a^ (n = 511), Honduras, August 29 to October 26, 2018** | | | |
| --- | --- | --- | --- |
|  | Nursing home | Daycare center |  |
|  | n = 182 | n = 329 |  |
| Variable | n (%) | n (%) | p-value^b^ |
| Sex |  |  | 0.017 |
| Female | 84 (46.1) | 188 (57.1) |  |
| Male | 98 (53.9) | 141 (42.9) |  |
| Age |  |  | <0.001 |
| ≤70 years | 45 (24.7) | 169 (51.4) |  |
| 71-80 years | 58 (31.9) | 111 (33.7) |  |
| ≥81 years | 70 (38.5) | 47 (14.3) |  |
| No data | 9 (4.9) | 2 (0.6) |  |
| Minimental test result^c^ |  |  | <0.001 |
| Normal | 120 (65.9) | 228 (69.3) |  |
| Slight deterioration | 9 (5.0) | 65 (19.7) |  |
| Moderate deterioration | 15 (8.2) | 22 (6.7) |  |
| Severe deterioration | 38 (20.9) | 14 (4.3) |  |
| Included in the survey | 95 (52.2) | 246 (74.8) |  |
| ^a^ Llamas-Velasco S, Llorente-Ayuso L, Contador I, Bermejo-Pareja F. Spanish versions of the Minimental State Examination (MMSE). Questions for their use in clinical practice. Revista de Neurologia. 2015 Oct;61(8):363-71. | | | |
| ^b^ P-value from Pearson Chi-square test. | | | |
| ^c^ We considered MMSE scores from 25-30 “normal,” 20-24 “slight deterioration,” 16-19 “moderate deterioration,” and ≤15 “severe deterioration. | | | |
