# Supplementary material for: Knowledge, attitudes, and practices of seasonal influenza vaccination among older adults in nursing homes and daycare centers, Honduras
Source: PLoS One. 2021 Feb 11;16(2):e0246382. doi: 10.1371/journal.pone.0246382 (PMC7877760; doi:10.1371/journal.pone.0246382)
Supplement: S2 Table — (DOCX) [file pone.0246382.s002.docx]

| **S2 Table. Demographics of 341 older adults stratified by recruitment location, Honduras, August 29 to October 26, 2018** | | | |
| --- | --- | --- | --- |
| Characteristic | Nursing home  (n = 95)  n (%) | Daycare center  (n = 246)  n (%) | p-value^a^ |
| Female sex | 47 (49.5) | 138 (56.1) | 0.271 |
| Age (in years) |  |  | <0.001 |
| 65-70 | 31 (32.6) | 125 (50.8) |  |
| 71-80 | 29 (30.5) | 86 (35.0) |  |
| ≥81 | 35 (36.9) | 35 (14.2) |  |
| Department of Residence |  |  | <0.001 |
| Francisco Morazán | 50 (52.6) | 56 (22.7) |  |
| Olancho | 17 (17.9) | 43 (17.5) |  |
| Lempira | 0 (0) | 59 (24.0) |  |
| La Paz | 0 (0) | 52 (21.1) |  |
| Choluteca | 5 (5.3) | 24 (9.8) |  |
| Other | 23 (24.2) | 12 (4.9) |  |
| Education (n = 330) |  |  | <0.001 |
| No formal education | 17 (18.1) | 69 (29.2) |  |
| Primary | 44 (46.8) | 155 (65.7) |  |
| ≥Secondary | 33 (35.1) | 12 (5.1) |  |
| Race |  |  | <0.001 |
| Mestizo | 85 (89.5) | 163 (66.3) |  |
| Indigenous | 2 (2.1) | 67 (27.2) |  |
| Other | 8 (8.4) | 16 (6.5) |  |
| Marital status |  |  | <0.001 |
| Married | 12 (12.6) | 92 (37.4) |  |
| Single | 42 (44.2) | 46 (18.7) |  |
| Accompanied | 1 (1.1) | 33 (13.4) |  |
| Separated/divorced | 14 (14.7) | 19 (7.7) |  |
| Widowed | 26 (27.4) | 56 (22.8) |  |
| Concurrent chronic disease^b^ | 71 (74.4) | 132 (53.7) | <0.001 |
| Self-reported influenza vaccination in 2017 (n = 331)^c^ | 66 (75.9) | 223 (91.4) | <0.001 |
| Self-reported influenza vaccination in 2018 (n = 329)^c^ | 62 (69.7) | 214 (89.2) | <0.001 |
| Verified^d^ influenza vaccination in 2018 (n = 284)^e^ | 43 (61.4) | 188 (87.9) | <0.001 |
| ^a^ P-value from Pearson Chi-square test. | | | |
| ^b^ 57 chronic heart disease, 42 diabetes mellitus, 14 asthma, 9 bronchitis, 8 chronic obstructive pulmonary disorder, 8 cerebrovascular disease, 3 cancer, 2 chronic kidney disease, 136 other disease. | | | |
| ^c^ Excluded participants who did not know if they were vaccinated. | | | |
| ^d^ Verified with vaccination cards and medical records. | | | |
| ^e^ Excluded 12 participants who did not know if they were vaccinated and 45 who professed vaccinations without verification. | | | |
