# Supplementary material for: Knowledge, attitudes, and practices of seasonal influenza vaccination among older adults in nursing homes and daycare centers, Honduras
Source: PLoS One. 2021 Feb 11;16(2):e0246382. doi: 10.1371/journal.pone.0246382 (PMC7877760; doi:10.1371/journal.pone.0246382)
Supplement: S3 Table — (DOCX) [file pone.0246382.s003.docx]

| **S3 Table. Knowledge of influenza vaccination stratified by recruitment location, older adults, Honduras, August 29 to October 26, 2018** | | | | | | | |
| --- | --- | --- | --- | --- | --- | --- | --- |
|  | Nursing home | | | Daycare center | | |  |
| Knowledge | Total^a^ n | Agreed  n | Agreed %  (95% CI) | Total^a^ n | Agreed  n | Agreed %  (95% CI) | p-value^b^ |
| Influenza causes severe illness | 91 | 83 | 91.2 (85.3–97.1) | 238 | 235 | 98.7 (97.3–100) | <0.001 |
| Older adults have a higher risk of complications from influenza | 91 | 87 | 95.6 (91.3–99.9) | 241 | 238 | 98.8 (97.3–100) | 0.075 |
| Influenza may be transmitted from person to person | 91 | 83 | 91.2 (85.3–97.1) | 237 | 230 | 97.0 (94.9–99.2) | 0.024 |
| Influenza may be transmitted if people touch their mouths or noses with contaminated hands | 94 | 85 | 90.4 (84.4–96.5) | 241 | 233 | 96.7 (94.4–99.0) | 0.020 |
| Aware of an influenza vaccine | 89 | 87 | 97.8 (94.6–100) | 243 | 242 | 99.6 (98.8–100) | 0.118 |
| The vaccine protects against influenza complications | 86 | 78 | 90.7 (84.4–96.7) | 238 | 235 | 98.7 (97.3–100) | <0.001 |
| Perceived vaccine as safe | 91 | 83 | 91.2 (85.3–97.1) | 240 | 233 | 97.1 (94.9–99.2) | 0.022 |
| CI: confidence interval | | | | | | | |
| ^a^ Excluded participants who did not respond. | | | | | | | |
| ^b^ P-value from Pearson Chi-square test. | | | | | | | |
