# Supplementary material for: Knowledge, attitudes, and practices of seasonal influenza vaccination among older adults in nursing homes and daycare centers, Honduras
Source: PLoS One. 2021 Feb 11;16(2):e0246382. doi: 10.1371/journal.pone.0246382 (PMC7877760; doi:10.1371/journal.pone.0246382)
Supplement: S4 Table — (DOCX) [file pone.0246382.s004.docx]

| **S4 Table. Associations between demographics and influenza vaccination (verified^a^), older adults in nursing homes (n = 63)^b^, Honduras, August 29 to October 26, 2018** | | | | |
| --- | --- | --- | --- | --- |
| Variable | OR (95% CI) | *P*-value | aOR^c^ (95% CI) | *P*-value |
| Female sex (Ref: male) | 0.77 (0.28–2.14) | 0.620 | – | – |
| Age (Ref: ≥81 years) |  | 0.618 |  |  |
| ≤70 years | 0.53 (0.15–1.89) |  | – | – |
| 71-80 years | 0.77 (0.23–2.58) |  | – | – |
| Education (Ref: ≥secondary) |  | 0.005 |  | 0.005 |
| No formal education | 14.00 (2.46–79.55) |  | 13.97 (2.03–96.00) |  |
| Primary incomplete or complete | 4.50 (1.31–15.42) |  | 8.70 (1.83–41.26) |  |
| Race (Ref: Mestizo) |  | 0.911 |  | – |
| Indigenous | – |  | – |  |
| Other | 0.61 (0.08–4.65) |  | – |  |
| Marital status (Ref: Single) |  | 0.224 |  | – |
| Married | 0.40 (0.08–2.00) |  | – |  |
| Accompanied | – |  | – |  |
| Separated / divorced | 0.83 (0.19–3.75) |  | – |  |
| Widowed | 2.89 (0.68–12.34) |  | – |  |
| Concurrent chronic disease (Ref: no) | 1.53 (0.52–4.50) | 0.443 | – | – |
| Self-reported influenza vaccination in previous year | 15.65 (3.06–80.19) | 0.001 | 23.18 (3.58–149.93) | 0.001 |
| Ref: reference; OR: odds ratio; aOR: adjusted odds ratio; CI: confidence interval | | | | |
| ^a^ Verified with vaccination cards and medical records. | | | | |
| ^b^ Analyses excluded participants with unverified influenza vaccinations in 2018, those who did not respond to educational attainment, and those who did not know their vaccination status in 2017. | | | | |
| ^c^ Adjusted for the other variables listed in the model. | | | | |
