# Supplementary material for: Knowledge, attitudes, and practices of seasonal influenza vaccination among older adults in nursing homes and daycare centers, Honduras
Source: PLoS One. 2021 Feb 11;16(2):e0246382. doi: 10.1371/journal.pone.0246382 (PMC7877760; doi:10.1371/journal.pone.0246382)
Supplement: S5 Table — (DOCX) [file pone.0246382.s005.docx]

| **S5 Table. Associations between demographics and influenza vaccination (verified^a^), older adults in daycare centers (n = 204)^b^, Honduras, August 29 to October 26, 2018** | | | | |
| --- | --- | --- | --- | --- |
| Variable | OR (95% CI) | *P*-value | aOR^c^ (95% CI) | *P*-value |
| Female sex (Ref: male) | 1.09 (0.45–2.64) | 0.855 | – | – |
| Age (Ref: ≥81 years) |  | 0.561 |  | – |
| ≤70 years | 1.90 (0.59–6.17) |  | – |  |
| 71-80 years | 1.61 (0.48–5.38) |  | – |  |
| Education (Ref: ≥secondary) |  | 0.870 |  | – |
| No formal education | 0.64 (0.07–5.71) |  | – |  |
| Primary incomplete or complete | 0.79 (0.10–6.60) |  | – |  |
| Race (Ref: Mestizo) |  | 0.184 |  | 0.112 |
| Indigenous | 2.46 (0.69–8.80) |  | 3.26 (0.84–12.71) |  |
| Other | 0.50 (0.13–1.99) |  | 0.49 (0.11–2.08) |  |
| Marital status (Ref: Single) |  | 0.222 |  | – |
| Married | 4.87 (1.19–19.96) |  | – |  |
| Accompanied | 1.20 (0.32–4.55) |  | – |  |
| Separated / divorced | 2.20 (0.24–19.90) |  | – |  |
| Widowed | 1.11 (0.36–3.49) |  | – |  |
| Concurrent chronic disease (Ref: no) | 1.43 (0.59–3.48) | 0.429 | – | – |
| Self-reported influenza vaccination in previous year | 7.21 (2.28–22.83) | <0.001 | 9.15 (2.66–31.42) | <0.001 |
| Ref: reference; OR: odds ratio; aOR: adjusted odds ratio; CI: confidence interval | | | | |
| ^a^ Verified with vaccination cards and medical records. | | | | |
| ^b^ Analyses excluded participants with unverified influenza vaccinations in 2018, those who did not respond to educational attainment, and those who did not know their vaccination status in 2017. | | | | |
| ^c^ Adjusted for the other variables listed in the model. | | | | |
