# Supplementary material for: Knowledge, attitudes, and practices of seasonal influenza vaccination among older adults in nursing homes and daycare centers, Honduras
Source: PLoS One. 2021 Feb 11;16(2):e0246382. doi: 10.1371/journal.pone.0246382 (PMC7877760; doi:10.1371/journal.pone.0246382)
Supplement: S6 Table — (DOCX) [file pone.0246382.s006.docx]

| **S6 Table. Reasons for receiving influenza vaccination stratified by recruitment location, older adults, Honduras, August 29 to October 26, 2018** | | | | | |
| --- | --- | --- | --- | --- | --- |
|  | Nursing home  (n = 43) | | Daycare center  (n = 188) | |  |
| Reason | Agreed  n | % (95% CI) | Agreed  n | % (95% CI) | p-value^a^ |
| *Easy access* |  |  |  |  |  |
| Offered the vaccine at nursing home or daycare center | 42 | 97.7 (93.0–100) | 80 | 42.6 (35.4–50.0) | <0.001 |
| Favorable vaccination hours | 5 | 11.6 (1.6–2.6) | 145 | 77.1 (71.1–83.2) | <0.001 |
| *Perceived benefits* |  |  |  |  |  |
| Perceived self-benefits of vaccination | 39 | 90.7 (81.7–99.7) | 166 | 88.3 (83.7–92.9) | 0.653 |
| Vaccine protects from complications | 21 | 48.8 (33.3–64.4) | 123 | 65.4 (58.6–72.3) | 0.043 |
| Perceived personal risk for influenza | 12 | 27.9 (13.9–41.9) | 93 | 49.5 (42.3–56.7) | 0.010 |
| Vaccination protects peers | 7 | 16.3 (4.8–27.8) | 30 | 16.0 (10.7–21.2) | 0.959 |
| Mild side effects perceived better than contracting influenza | 14 | 32.6 (18.0–47.1) | 87 | 46.3 (39.1–53.5) | 0.102 |
| To negate costs of treatment for influenza | 17 | 39.5 (24.3–54.8) | 118 | 62.8 (55.8–69.7) | 0.005 |
| *Previous experiences* |  |  |  |  |  |
| No problems with previous vaccination | 20 | 46.5 (31.0–62.0) | 107 | 56.9 (49.8–64.1) | 0.216 |
| Have not observed negative effects of vaccination | 13 | 30.2 (15.9–44.5) | 41 | 21.8 (15.9–27.8) | 0.239 |
| *Peer influence* |  |  |  |  |  |
| Peers recommended vaccination | 12 | 27.9 (13.9–41.9) | 41 | 21.8 (15.9–27.8) | 0.391 |
| Knowledge that the majority of peers get vaccinated | 11 | 25.6 (12.0–39.2) | 33 | 17.6 (12.1–23.0) | 0.227 |
| Peers expected vaccination | 7 | 16.3 (4.8–27.8) | 19 | 10.1 (5.8–14.5) | 0.248 |
| Urged to get vaccinated by family members | 2 | 4.7 (0–11.2) | 64 | 34.0 (27.2–40.9) | <0.001 |
| *Health establishment counseling* |  |  |  |  |  |
| Was informed vaccination is mandatory | 8 | 18.6 (6.5–30.7) | 71 | 37.8 (30.8–44.8) | 0.017 |
| Urged to get vaccinated by a doctor or nurse | 9 | 20.9 (8.3–33.6) | 134 | 71.3 (64.7–77.8) | <0.001 |
| Listened to promotional outreach on vaccinations at nursing home or daycare center | 7 | 16.3 (4.8–27.8) | 39 | 20.7 (14.9–26.6) | 0.508 |
| *Aware of vaccine benefits from mass media* | 4 | 9.3 (0.3–18.3) | 64 | 34.0 (27.2–40.9) | <0.001 |
| CI: confidence interval | | | | | |
| ^a^ P-value from Pearson Chi-square test. | | | | | |
