# Supplementary material for: Knowledge, attitudes, and practices of seasonal influenza vaccination among older adults in nursing homes and daycare centers, Honduras
Source: PLoS One. 2021 Feb 11;16(2):e0246382. doi: 10.1371/journal.pone.0246382 (PMC7877760; doi:10.1371/journal.pone.0246382)
Supplement: S1 Questionnaire — (DOCX) [file pone.0246382.s007.docx]

**Encuesta a adultos mayores**

[*Nota: este formulario no se utilizará impreso, solo en tableta*]

1. Nombre del establecimiento: ____________________________________
2. Fecha de la entrevista: ____/_____/_____ (dd/mm/aaaa)
3. Código para el estudio: _____________________________________________
4. Nombre de la persona entrevistada: ____________________________________
5. Sexo:  Masculino;  Femenino
6. ¿Cuántos años cumplidos tiene? ________ años.
7. ¿Cuál es su fecha de nacimiento? ____/_____/_____ (dd/mm/aaaa)
8. Departamento donde reside: _________________
9. Municipio donde reside: ___________________
10. ¿Hasta qué nivel de estudios ha llegado usted?:

No sabe leer ni escribir, y no ha realizado estudios formales

Sabe leer y escribir, pero no ha realizado estudios formales

Primaria incompleta

Primaria completa

Secundaria incompleta

Secundaria completa

Bachillerato o diversificado incompleto

Bachillerato o diversificado completo

Estudios universitarios

Maestría profesional, especialización o postgrado

Doctorado

No responde

1. ¿Con cuál de las siguientes etnias usted se identifica?

Blanca;

Mestiza;

Mulata;

Descendiente africana;

Indígena

Especificar:

1. Lenca,
2. Garífuna,
3. Tolupanes,
4. Misquitos,
5. Pech,
6. Chortís,
7. Tawahka,
8. Otra categoría indígena (p10h1) ¿Cuál? __________)

Otra etnia (p10i) ¿Cuál? __________

No sabe,

No responde.

1. ¿Cuál es su estado actual civil? [leer opciones en voz alta]

Soltero o soltera (nunca casado o acompañado)

Casado o casada

Acompañado o acompañada

Divorciado o divorciada

Separado o separada

Viudo o viuda

No responde

**En la siguiente sección le leeremos sobre la influenza o gripe y la vacunación contra esta enfermedad. Le pedimos que nos diga si usted está de acuerdo con lo que leímos o no esta de acuerdo. También puede decirnos que no sabe, o que no desea responder.**

1. La influenza o gripe puede causar una enfermedad grave.

De acuerdo;  En desacuerdo;  No sabe  No responde

1. Todas las personas están en riesgo de enfermar por influenza, pero los adultos mayores tienen más chance de complicarse (ejemplo hospitalización o muerte) .

De acuerdo;  En desacuerdo;  No sabe  No responde

1. La influenza o gripe puede pasarse entre las personas

De acuerdo;  En desacuerdo;  No sabe  No responde

1. Las personas pueden enfermar por influenza o gripe si toca su boca o nariz después de haber tocado algo contaminado o ensuciado liquidos de personas enfermas de gripe.

De acuerdo;  En desacuerdo;  No sabe  No responde

1. Existe una vacuna para evitar la influenza o gripe

De acuerdo;  En desacuerdo;  No sabe  No responde

1. La vacuna contra influenza o gripe puede proteger a una persona contra la influenza y sus complicaciones.

De acuerdo;  En desacuerdo;  No sabe  No responde

1. Es seguro para usted vacunarse contra la influenza o gripe.

De acuerdo;  En desacuerdo;  No sabe  No responde

**Ahora le voy a hacer unas preguntas sobre la vacunación contra la influenza o gripe**

1. ¿Fue usted fue vacunado(a) contra la influenza el año pasado?

Sí  No  Planee vacunarme, pero no pude  No sabe  No responde

1. ¿Fue usted fue vacunado(a) contra la influenza en lo que va de este año?

Sí  No  No sabe  No responde Si es No,

1. De las siguientes opciones, ¿Cuál o cuáles son los motivos por lo que no se vacunó (*o no se vacunará si aun no ha finalizado la campaña de vacunación del 2018*) en la campaña de 2018? (puede marcarse más de una) [*esta pregunta se hará en caso que aun no haya finalizado la campaña de vacunación*]

Se orientará al encuestador para que pregunte los motivos por los qué el adulto mayor decidió no vacunarse. Luego marcará todos los motivos que el adulto mayor le dijo. Inmediatamente después preguntará sobre los ítems que no se marcaron, por si hay algún otro que olvidó. El encuestador podrá agrupar las presuntas de rechazo a la vacunación. En caso admita el motivo general, se preguntarán los motivos listados.

¿Tiene miedo de la vacuna?

1. Tiene miedo a enfermar de gripe si se pone la vacuna contra la influenza
2. Tiene miedo de la calentura, picazón, dolor y otras cosas que aparecen después de vacunarse
3. Tiene miedo al dolor que causa la aguja

¿Cree que la vacuna no es buena?

1. Cree que la vacuna no es buena para evitar la enfermedad
2. No cree que pueda enfermarse gravemente por influenza (no vale la pena vacunarse)
3. No cree que el que usted se vacune evite que sus compañeros en este Centro de Atención del Adulto Mayor enfermen por influenza

¿Sabe dónde ir a vacunarse?

1. Fue al centro de salud a vacunarse contra la influenza, pero no había vacuna disponible
2. Nadie le dijo que debía vacunarse contra la influenza
3. No se le ha ofrecido la vacuna
4. No sabe a dónde ir por una vacuna
5. La vacuna es demasiado cara

¿Le han dicho que no se vacune?

1. La vacunación contra la influenza no es aceptada por los demás compañeros en el Centro de Atención del Adulto Mayor
2. Sus familiares le dijeron que no se vacunara
3. Sus amigos le dijeron que no se vacunara
4. Otro (p20o1) ¿Cuál?_______________________________________________
5. ¿Cuál fue el motivo por el que ha decidido vacunarse contra la influenza?

Se orientará al encuestador para que pregunte los motivos por los qué el adulto mayor decidió vacunarse. Luego marcará todos los motivos que el adulto mayor le dijo. Inmediatamente después preguntará sobre los ítems que no se marcaron, por si hay algún otro que olvidó. El encuestador podrá agrupar las presuntas de por qué se vacunó. En caso admita el motivo general, se preguntarán los motivos listados.

¿Le fue fácil vacunarse?

1. Me ofrecieron la vacuna en el Centro de Atención del Adulto Mayor
2. Los horarios de vacunación son buenos para mi

¿Cree que es necesario vacunarse?

1. Las vacunas son buenas para mí
2. Creo que la vacuna puede protegerme de enfermar gravemente de infleunza
3. Considero que soy una persona en riesgo de enfermar gravemente por influenza, como ser hospitalizado o morir a debido a ella
4. Al vacunarme evito pasar la enfermedad a mis compañeros en al Centro de Atención del Adulto Mayor

¿Considera que la vacuna no es mala?

1. Creo que los efectos adversos de la vacuna contra la influenza o gripe no son tan malos en comparación a las complicaciones que podría ocasionarme el enfermar por influenza
2. Prefiero ponerme la vacuna que gastar en el tratamiento por la enfermedad (influenza)
3. Antes me vacuné contra la influenza y no me pasó nada malo
4. No he visto que le haya sucedido algo malo a algún familiar o compañero por vacunarse contra la influenza

¿Le han dicho que debe vacunarse?

1. Me dijeron que era obligación que me vacunara contra la influenza
2. Mis compañeros en el Centro de Atención del Adulto Mayor me recomendaron que me vacunara contra la influenza
3. La mayoría de mis compañeros en este Centro de Atención del Adulto Mayor se vacunaron contra la influenza
4. Mis compañeros en el Centro de Atención del Adulto Mayor esperan que yo me vacune contra la influenza
5. Mis familiares me pidieron que me vacunara contra la influenza
6. Un médico o una enfermera me recomendaron que me vacunara contra la influenza
7. Nos dieron una platica en el Centro de Atención del Adulto Mayor dónde se nos aconsejaba que nos vacunáramos contra la influenza.
8. Me di cuenta (en la radio, televisión, periódico, internet u otro medio masivo de comunicación) que era bueno que me vacunara contra la influenza
9. Otro motivo. (p23s1) ¿Cuál?_________________________________________
10. ¿Presentó algún malestar en los 7 días posteriores a la vacunación del 2018?

Sí  No  No recuerda  No responde

Si es Sí:

1. ¿Cuál síntoma presentó?
2. Malestar general;
3. Dolor en el sitio de la vacunación;
4. Hinchazón y/o enrojecimiento en el sitio de vacunación
5. Hematoma en el sitio de vacunación
6. Urticaria
7. Reacción alérgica
8. Fiebre o sensación de fiebre;
9. Mareos
10. Síntomas similares a una gripe;
11. Otro. (p25j1) ¿Cuál? ___________________
12. No sabe
13. No responde
14. Fecha de vacunación contra la influenza (fecha verificada): _____/_____/______ (dd/mm/aaaa)
15. Fuente de información:
16. Carné de vacunación
17. Expediente médico
18. Llamada telefónica
19. Visita centro de vacunación
20. No se logró verificar la fecha de la vacunación.

**Muchas gracias por haber participado en esta encuesta.**

**Se complementará la encuesta con la información del expediente del adulto mayor:**

1. En el expediente médico del adulto mayor está anotada algunas de las siguientes enfermedades crónicas:

| **Enfermedad** | **Sí** | **No** | **Sin dato** |  | **Enfermedad** | **Sí** | **No** | **Sin dato** |
| --- | --- | --- | --- | --- | --- | --- | --- | --- |
| 1. Enf. Crónica del corazón |  |  |  |  | 1. Diabetes mellitus |  |  |  |
| 1. Asma |  |  |  |  | 1. Enf. Renal crónica |  |  |  |
| 1. Bronquitis |  |  |  |  | 1. Inmunodepresión (incluye VIH) |  |  |  |
| 1. EPOC |  |  |  |  | 1. Cáncer |  |  |  |
| 1. Fibrosis quística |  |  |  |  | 1. Enfermedad cerebrovascular |  |  |  |

p28k Otra:  Sí;  No (p28k1)¿Cuál? ___________________________________________

**Survey in English**

1. Healthcare facility name: ____________________________________
2. Date of interview: ____ / _____ / _____ (dd / mm / yyyy)
3. Code assigned to the participant: _____________________________________________
4. Name of the interviewee: ____________________________________
5. How old are you? ________ years.
6. What is your birth date? ____ / _____ / _____ (dd / mm / yyyy)
7. Department of residence: _________________
8. Municipality where you reside: ___________________
9. Highest level of education completed?:

Cannot read or write / no formal education studies

Literate, but has no formal education

incomplete primary

full primary

incomplete secondary

Completed secondary

Diversified secondary education or incomplete

Diversified secondary education or full

university

professional expertise, specialization or postgraduate

Doctorate

no response

1. Which of the following ethnic groups you identify?

white;

mestizo;

Mulata;

African descendent;

Indigenous

Specify:

1. Lenca,
2. Garifuna,
3. Tolupanes,
4. Miskito,
5. Pech,
6. Chorti,
7. Tawahka,
8. Another Indian category (p10h1) What? __________)

Another ethnic group (p10i) What? __________

Does not know,

No response.

1. What is your current marital status? [Read options aloud]

Single (never married or accompanied)

Married

Accompanied

Divorced

Separated

Widowed

no response

**In the following section we will read a series of assertions on influenza and their vaccination. We ask that you tell us whether you agree or disagree with each. You can also tell us that you do not know, or do not want to respond.**

1. Influenza causes severe illness.

Agree;  In disagreement;  Does not know  no response

1. Older adults have a higher risk of complications from influenza.

Agree;  In disagreement;  Does not know  no response

1. Influenza may be transmitted from person to person

Agree;  In disagreement;  Does not know  no response

1. Influenza may be transmitted if people touch their mouths or noses with contaminated hands

Agree;  In disagreement;  Does not know  no response

1. There is a vaccine to prevent influenza or flu

Agree;  In disagreement;  Does not know  no response

1. The vaccine protects against influenza complications

Agree;  In disagreement;  Does not know  no response

1. The influenza vaccine is safe for you.

Agree;  In disagreement;  Does not know  no response

**Now I'll ask you some questions about vaccination against influenza**

1. Were you vaccinated against influenza during 2017 vaccination campaign?

Yes  No  Does not know  no response

1. Were you vaccinated against influenza during 2018 vaccination campaign?

Yes  No  Does not know  no response

1. Of the following options, what or what are the reasons because you was not vaccinated (or not be vaccinated if it has not yet completed the vaccination campaign of 2018) in the 2018 campaign? (Can be marked more than one) [this question will be asked even if it has not completed the vaccination campaign]
2. Fear of contracting influenza
3. Fear of side effects
4. Fear of pain caused by the needle
5. Belief vaccine does not prevent disease
6. Belief that influenza is not serious
7. Belief vaccine does not protect peers
8. Vaccine not available
9. Was not instructed to be vaccinated
10. Was not offered the vaccine
11. Did not know where to go for vaccine
12. The vaccine is too expensive
13. Vaccine not accepted by nursing home or daycare center peers
14. Family members said not to get vaccinated
15. Friends said not to get vaccinated
16. Other (p20o1) What? _______________________________________________
17. What was the reason you decided to get vaccinated against influenza?
18. Offered the vaccine at nursing home or daycare center
19. Favorable vaccination hours
20. Was informed vaccination is mandatory
21. Perceived self-benefits of vaccination
22. Vaccine protects from complications
23. Perceived personal risk for influenza
24. Vaccination protects peers
25. Mild side effects perceived better than contracting influenza
26. To negate costs of treatment for influenza
27. No problems with previous vaccination
28. Have not observed negative effects of vaccination
29. Peers recommended vaccination
30. Knowledge that the majority of peers get vaccinated
31. Peers expected vaccination
32. Urged to get vaccinated by family members
33. Urged to get vaccinated by a doctor or nurse
34. Listened to promotional outreach on vaccinations at nursing home or daycare center.
35. Aware of vaccine benefits from mass media
36. Another reason. (P23s1) What? _______________________________________
37. Did you file any symptoms within 7 days after vaccination in 2018?

Yes  No  Does not remember  no response

If yes:

1. What symptom presented?
2. General discomfort;
3. Pain at the site of vaccination;
4. Inflammation at the site of vaccination
5. Hematoma at the site of vaccination
6. Urticaria
7. Allergic reaction
8. Fever or feeling of fever;
9. lightheadedness
10. flu-like symptoms;
11. Other. (P25j1) What? ___________________
12. Does not know
13. no response
14. Date of influenza vaccination (verified date): _____ / _____ / ______ (dd / mm / yyyy)
15. Source of information:
16. Vaccination card
17. medical record
18. Phone call
19. Visit vaccination center
20. It was not possible to verify the date of vaccination.

**Thank you very much for participating in this survey.**

**The survey was supplemented by information on the medical record:**

1. In the medical record of the elderly it is annotated some of the following chronic diseases:

| **Disease** | **Yes** | **No** | **No data** |  | **Disease** | **Yes** | **No** | **No data** |
| --- | --- | --- | --- | --- | --- | --- | --- | --- |
| 1. Chronic heart disease |  |  |  |  | 1. diabetes mellitus |  |  |  |
| 1. Asthma |  |  |  |  | 1. chronic renal disease |  |  |  |
| 1. Bronchitis |  |  |  |  | 1. Immunosuppression (including HIV) |  |  |  |
| 1. COPD |  |  |  |  | 1. Cancer |  |  |  |
| 1. Cystic fibrosis |  |  |  |  | 1. cerebrovascular disease |  |  |  |

p28k Other:  Yes; No (p28k1) What? ___________________________________________
